# Therapeutic Approaches to the Alternative Angiotensin-(1–7) Axis of the Renin-Angiotensin System

**Published:** 2017-11-29

**Authors:** Mark C Chappell

**Affiliations:** Department of Surgery, Hypertensin and Vascular Research, Wake Forest School of Medicine, USA

## Abstract

Cardiovascular disease remains the leading cause of death for both men and women in the United States despite the recent advances in drug development, changes in lifestyle and screening protocols. A key target in the treatment of cardiovascular disease and hypertension is the renin-angiotensinsystem (RAS), a circulating and tissue system involved in the regulation of blood pressure, fluid balance and cellular injury. Pharmacologic approaches have traditionally focused on the Ang II-AT1receptor axis of the RAS to prevent the generation of Ang II with angiotensin converting enzyme inhibitors (ACEI) or to block the binding of Ang II to the AT1 receptor (AT1R) with selective antagonists (ARBs).

## Introduction

Beginning with our demonstration of endogenous levels of the heptapeptide peptide Ang-(1–7) in the circulation, brain and other relevant cardiovascular tissues, alternative pathways within the RAS are present that generate bioactive peptides other than Ang II [1]. The concept of an alternative RAS with Ang-(1–7) as the principal bioactive component was bolstered by identification of the Mas receptor that mediates the actions of Ang-(1–7) by Santos, Bader, Thomas and colleagues, as well as the ACE homolog ACE2 capable of directly converting Ang II to Ang-(1–7)by the Acton, Turner, and Penninger labs [2–5]. Ang-(1–7) exhibits distinct actions to functionally oppose the deleterious effects of an activated ACE-Ang II-AT1R axis [6]. Experimental evidence to date suggests that Ang-(1–7) exhibits a wide range of cardioprotective effects that involve the stimulation of the nitric oxide synthase (NOS) and release of NO, the attenuation of oxidative stress and a reduction in inflammatory signaling [7]. The current review examines the various approaches that would target the alternative Ang-(1–7) axis in the treatment of cardiovascular disease and other pathologies including cancer, diabetes and tissue fibrosis [7].

## Angiotensin-(1–7) Formation

Circulating Ang-(1–7) is derived from the inactive precursor peptide Ang I by the metalloendopeptidase (MEP) neprilysin (EC 3.4.24.11) [8]. Although there is little to no circulating neprilysin, the endopeptidase is anchored to the cell membrane with the active site orientated to the extracellular space that allows for the processing of circulating Ang I to Ang-(1–7). Apart from the circulation, Ang-(1–7) expression is evident in many tissues and may reflect the processing of Ang I by an intracellular MEP - thimet oligopeptidase (EC 3.4.24.15) [8]. Both endopeptidases efficientlyhydrolyze the Pro7-Phe8 bond of Ang I to directly generate Ang-(1–7); however, neprilysin also cleaves of the Tyr4-Ile5 of Ang I to form Ang-(1–4) [8].Nagata and colleagues have identified an extended form of Ang I termed Ang-(1–12) in plasma and various tissues in rat that may reflect renin-independent processing of the RAS precursor angiotensinogen [9]. We originally found that Ang-(1–12) is also a substrate for neprilysin to generate Ang-(1–7); however, Ang-(1–12) is converted to Ang II by successive hydrolysis of Tyr10-Tyr11 and Phe8-His9 bonds via ACE [10]. This is likely the predominant processing pathway for Ang-(1–12) in the circulation given the high levels of ACE and that the increase in blood pressure to Ang-(1–12) infusion was abolished by an ACE inhibitor [9]. Endogenous levels of Ang-(1–7) may also originate from processing of Ang II by the mono-carboxypeptidasesangiotens in converting enzyme 2 (ACE2, EC3.4.17.23). Prolyl oligopeptidase (EC3.4.21.26) and prolyl carboxypeptidase (EC3.4.16.2)[8]. These path ways initially require the traditional processing of Ang I by ACE to generate Ang II as the substrate for the conversion to Ang-(1–7). Santos and colleagues also identified an endogenous isoform of Ang-(1–7) in human plasma that arises from decarboxylation of the aspartic acid residue to alanine to form [Ala1]-Ang-(1–7) [11]. The peptide appears to share similar properties to Ang-(1–7); however, [Ala1]-Ang-(1–7) may preferentially bind to the Mas-related G protein-coupled receptor D (MRG-D) rather than the MasR. Moreover, both the AT7/MasR antagonist [D-Pro7]-Ang-(1–7) and the AT2R antagonist PD123319, but not [D-Ala7]-Ang-(1–7) block the vasorelaxant effects of the peptide [11]. It is presently unknown whether Ang-(1–7) itself is decarboxylated or that existing alanine isoforms of Ang II and Ang I are processed to [Ala1]-Ang-(1–7).

## Ace2

ACE2 is an extracellularly-oriented monocarboxypeptidase that converts Ang II to Ang-(1–7) and the enzyme is widely distributed in various tissues [8]. We previously showed that ACE2 was the primary enzymatic pathway in the mouse heart for the formation of Ang-(1–7) from Ang II consistent with studies in the human heart [12–13].The peptidase is now considered a key target of the RAS as the altered expression of the enzyme may influence the balance of Ang II and Ang-(1–7) [8] Raizada and colleagues initially recognized the importance of this regulatory relationship and identified two allosteric activators of ACE2 (xanthenone and diminazene aceturate) that increase peptidase activity about 2-fold [14]. Administration of these small molecule activatorssignificantly reduced blood pressure and attenuated tissue fibrosis; these effects were reversed by [D-Ala7]-Ang-(1–7) antagonist suggesting a primary action of Ang-(1–7) rather than Ang II [15–17]. However, the cardioprotective effects of ACE2 activators have not been consistently replicated by other investigators which may in part reflect different models of cardiovascular disease or tissue injury [18–20]. Moreover, it is now apparent that these activators may exhibit other properties that are distinct from an allosteric effect to augment the catalytic rate of ACE2 [18–20].

An alternative approach to pharmacologically modifying ACE2 activity is the direct administration of a soluble form of recombinantACE2 (rACE2) that retains the specificity and catalyticactivity of the native enzyme [21–22]. The premise of ACE2 supplementation is that sufficiently high levels of ACE2 are administered to reduce Ang II/Ang-(1–7) thereby attenuating the actions of Ang II-AT1R axis while amplifying the Ang-(1–7)-AT7/MasR effects (Figure 1). Oudit and colleagues find that chronic administration of soluble rACE2 attenuated various indices of cardiac and renal injury; inflammation and fibrosis in both type 1 and type 2 diabetic mice [23–24]. Surprisingly, the administration of rACE2 reduced tissue levels of Ang II in the heart and kidney and increased the tissue contents of Ang-(1–7) [23]. It is unclear how increased circulating rACE2 augments Ang-(1–7) tissue levels as the intracellular mechanism for Ang II or Ang-(1–7) generation is not known. One possibility is that administered rACE2 increases circulating levels of Ang-(1–7) and the peptide is subsequently internalized by the Mas receptor into a stable or protected intracellular compartment. In turn, rACE2 treatment may reduce circulating levels of Ang II that would lead to less internalization of the Ang II-AT1R complex and reduced tissue content. Alternatively, rACE2 treatment may alter that intracellular signaling milieu to attenuate oxidative stress or inflammation that impacts the local generation of Ang II and Ang-(1–7). Scholey and colleagues also report that rACE given subcutaneously by an osmotic pump attenuated several indices of renal damage in the transgenic Col4A3−/− mouse, a model of Alport syndrome, as well as tended to lower blood pressure [25]. The renal protective effects of soluble rACE2 treatment were associated with a marked reduction in the ratio of Ang II to Ang-(1–7) in the kidney have (from 69 to 15) while the wild type Ang II/Ang-(1–7) value was 4 (peptide values were corrected to fmol/mg protein for ratio determination) [25]. However, Wysocki et al observed that neither the administration of rACE2 nor the chronic over expression of the soluble peptidase by minicircle DNA conveyed any protective effects against diabetic nephropathy in diabetic mice [26]. In this study, the plasma angiotensin peptides were quantified by UHPLC-mass spectroscopy with sufficient sensitivity to detect peptides in the low pM range and resolve related N-terminal metabolites of Ang I, Ang II and Ang-(1–7) that include Ang-(2–10), Ang-(2–8), Ang-(3–8), Ang-(2–7) and Ang-(3–7), respectively; these metabolites are not distinguished by direct RIAs or ELISAs unless coupled to HPLC/UHPLC separation prior to assay. This analysis revealed that chronic ACE2 treatment reduced the total plasma ratio of Ang II to Ang-(1–7) approximately 4–fold in the diabetic mice; however, the effect of ACE2 on renal peptide content was not addressed. Moreover, the fact that the N-terminal metabolites Ang-(2–10), Ang-(2–8) or Ang III and Ang-(2–7) were the major components in plasma contrasts with the accepted profile of angiotensins in both the circulation and tissues [8].

Although the disparate findings of rACE2 treatment to ameliorate renal injury are difficult to reconcile, the concept of ACE2 administration to regulate the endogenous RAS may present several disadvantages. One concern is that a reduction in Ang II and the subsequent lowering of blood pressure by exogenous ACE2 should markedly stimulate the conventional RAS as the negative feedback mechanisms on reninare removed or disinhibited. Elevated renin will generate Ang I, and given the very high ACE content in the vasculature, Ang I is immediately converted to Ang II to increase the peptide. The second issue is since ACE is the major pathway for the metabolism of Ang-(1–7), it appears unlikely that exogenous ACE2 would result in a sustained increase in Ang-(1–7) unless ACE activity is sufficiently blocked or down regulated ACE readily hydrolyzes the Ile5-His6bond of Ang-(1–7) to generate the dipeptide His-Pro and Ang-(1–5) which explains the increased circulating levels of Ang-(1–7) with ACE inhibitors [8]. We suggest that a more effective approach would be the administration of ACE2 combined with an ACE inhibitor to enhance the metabolism of residual levels of Ang II and prevent the degradation of the Ang-(1–7). Importantly, this approach would not impact the endogenous pathway for the conversion of Ang I [or Ang-(1–12)] to Ang-(1–7) by neprilysinor thimet oligopeptidase as the Ang II substrate is depleted.

## Angiotenisn-(1–7) Analogs

An alternative approach to increase endogenous levels of Ang-(1–7) by ACE2 is the direct administration of the peptide. However, the native peptide is rapidly metabolized by ACE in the circulation and cannot be administered orally. To circumvent the bioavailability issue, investigatorsat Aventis Pharmaceutical synthesized the first non-peptide agonist (AVE 0991) to the AT7/MasR that is orally active and is likely not a substrate of ACE [27]. AVE0991 appears to share the same properties of Ang-(1–7) and the actions of the analog are blocked by the AT7/MasR antagonists [D-Ala7]-Ang-(1–7) and [D-Pro7]-Ang-(1–7) [28]. Santos and colleagues have developed a different approach in which Ang-(1–7) is encapsulated within a cyclodextrin complex that is orally available and conveys similar biological actions as Ang-(1–7), although it’s unclear whether the cyclodextrin form of Ang-(1–7) is more resistant to metabolism than the native peptide [29]. Haas and colleagues synthesized a peptide analog of Ang-(1–7) in which the peptide contained a thioether bridge between the substituted residues Ser4 and Cys6 [30]. This peptide analog is biologically active and is protected from ACE hydrolysis by the bridged residue at the 6th position [30–31]. We recently reported the effects of single substitutions along the Ang-(1–7) peptide chain using novel non-natural δ-amino acids derivatives (ACCA) on peptide metabolism and inhibition of cell growth. ACCA substitutions to Iso5 or His6 of Ang-(1–7) essentially abolished the metabolism of the peptide analog by human ACE, as well as retained the anti-proliferative actions of the peptide in two tumor cell lines [32]. Interestingly, the ACCA substitution of Val3did not prevent ACE metabolism, but did abolish N-terminal hydrolysis by dipeptidyl aminopeptidase 3 (EC3.4.14.4; DAP 3). DAP 3 hydrolyses Ang-(1–7) to Ang-(5–7) in a 2-step reaction that initially generates the dipeptide Asp-Arg and Ang-(3–7), and then rapidly cleavesAng-(3–7) to the dipeptide Val-Tyr and the tripeptide Ile-His-Pro [33]. We previously reported that DAP 3 activity was increased 3-fold in the cerebrospinal fluid (CSF) of adult sheep that were exposed to betamethasone in utero as a model of fetal programing to hasten pulmonary development of premature infants [7]. DAP 3 activities in the CSF increased to a greater extent than ACE in the betamethasone-exposed sheep and both peptidases may contribute to the lower CSF levels of Ang-(1–7) and an altered baroreflex control of blood pressure [7]. DAP 3 activity was also evident in the intracellular and extracellular compartments of human HK-2 proximal tubule cells [34]. In these cells, treatment with the MEP inhibitor JMV-390 attenuated DAP 3 activity and increased the intracellular levels of Ang-(1–7) approximately 2-fold; however, a higher concentration of JMV-390 significantly reduced Ang-(1–7) in the HK-2 cells that likely reflects crossover of the inhibitor to block thimet oligopeptidaseto reduce Ang-(1–7) generation [33].

Finally, we note that a non-peptide orally available agonist of the AT2R (C21) was recently developed and exhibits cardioprotective effects similar to the Ang-(1–7) pathway. Katovitchand colleagues find that C21 attenuated the extent of pulmonary fibrosis and pulmonary hypertension [35]. These protective effects of C21 were abolished by the AT2R antagonist PD123319; however, the C21 effects were also blocked by the [D-Ala7]-Ang-(1–7) antagonist [35]. It is presently unclear whether the C21 compound directly stimulates the AT7/MasR or that activation of the AT2R requires the interaction or transactivation of the Ang-(1–7) receptor; however, both the AT2R and AT7/MasR were required to transduce the effects of XX to inhibit proliferation of glial cells [36]. We find that the potent vasorelaxation of the renal artery by Ang-(1–7) (ED50 of 3 nM) was blocked by [D-Ala7]-Ang-(1–7) and the AT2R antagonist PD123319, as well as the soluble guanylate cyclase inhibitor ODQ [37]. Thus, it is feasible that the AT2R agonist C21 may also stimulate the Ang-(1–7)-AT7/MasR axis and that C21 could be combined with conventional RAS therapies including ACEIs or ARBs.

## Conclusion

In conclusion, activation of the Ang-(1–7)-AT7/MasR axis may be a potentially important therapeutic target in the treatment of cardiovascular disease. The continued development of selective non-peptide analogs of Ang-(1–7) that target both Mas and MRG-D receptors may be of particular benefit in terms of their oral availability, greater resistance to peptidase metabolism and their potential to accumulate within the cell. Indeed, we demonstrated the expression of intracellular AT7/Mas receptors on isolated nuclei from the ovine kidney that were linked to NO generation [38]. Moreover, the AT7/MasR binding density on renal nuclei and the Ang-(1–7)-evoked NO response was reduced in aged sheep and those exposed to betamethasone in utero as compared to the younger non-exposed animals [7,38]. Conversely, intracellular levels of the AT1R that were associated with stimulation of oxidative stress on isolated nuclei were increased in the older animals and in the betamethasone-exposed sheep [7, 38]. These latter findings of nuclear AT1R on the ovine renal cortex support extensive evidence for an intracellular RAS within various tissues although the exact role of an activated intracellular RAS to influence cardiovascular disease and other pathologies is unknown and awaits more conclusive studies [38–40].Nevertheless, peptidase resistant and cell permeable agonists of the Ang-(1–7) axis may provide additional cardioprotective effects to conventional approaches to block the RAS.
